# Supplementary material for: Knowledge, attitudes and practices towards COVID-19 among healthcare workers: A cross-sectional survey from Kiambu County, Kenya
Source: PLoS One. 2024 Mar 12;19(3):e0297335. doi: 10.1371/journal.pone.0297335 (PMC10931472; doi:10.1371/journal.pone.0297335)
Supplement: S1 Table — This is a summary table of all the KAP crude odds ratio and their respective p-values used to construct the initial fitted model for ordinal logistic regression. (PDF) [file pone.0297335.s002.pdf]

S2 Table. Crude odds ratio and the p-values. This is a summary table of all the KAP crude odds ratio and their respective p-values used to construct the initial fitted model for ordinal logistic regression.

**Target variable**	**Predictor variable**	**COR**	**p-value**
**Knowledge**			
**Sex**			
	Female	Ref	
	Male	1.062	0.615
	Prefer not to say	0.257	**0.015**
**Age**			
	18-24	Ref	
	25-29	0.6200790	0.47110363
	age30-34	0.6074125	0.45033384
	age35-39	0.4499865	0.22617238

	age40-44	0.3504327	0.11311214
	age45-49	0.5761222	0.41177033
	age50-54	0.5710036	0.41492236
	age55-59	0.6547667	0.53411557
Work place			
	County official	Ref	
	Level 1		0.47110363
	2		0.45033384
	3		0.22617238
	4		0.11311214
	5		0.41177033
	Sub-county		0.41492236
**Education**			

	Bachelors	Ref	
	Certificate	0.6719	0.0855
	**Diploma**	**0.5005**	**0.0000**
	Masters	0.9913	0.9705
	Other	0.4048	0.1043
	PhD	0.0020	0.9832
**Cadre**			
	Administrative staff	Ref	
	**Caregivers**	**0.5966**	**0.00180**
	Environmental health staff	1.044	0.8486
**Sources of information**			
	Government	0.938	0.6770
	**News**	**1.458**	**0.00117**

	**International**	**1.593**	**0.000052**
	**Social media**	**1.8811**	**0.00000006**
	**Continuous medical fora**	**3.750**	**0.00000000000000008**
	**Journals**	**2.391**	**0.00000003**

**Target variable**	**Predictor variable**	**COR (C.I.)**	**p-value**
**Attitude**			
**Sex**			
	Female	Ref	
	Male	0.8843	0.3649
	Prefer not to say	1.4796	0.5483
**Age**			
	18-24	Ref	
	25-29	0.7176120	0.5269
	age30-34	0.6827618	0.4669
	age35-39	1.0365418	0.9450
	age40-44	1.1028746	0.8511
	age45-49	0.8816139	0.8156

	age50-54	0.8066674	0.7129
	age55-59	0.8400902	0.7514
**Work place**			
	Level 1	1.5102	0.4467
	2	2.0978	0.05007
	3	1.6489	0.1630
	4	0.8639	0.6792
	5	1.1205	0.7527
	Sub-county	1.0628	0.8896
	County official	Ref	
**Education**			
	Bachelors	Ref	
	Certificate	1.629	0.0560

	**Diploma**	**1.2557**	0.1836
	Masters	0.9629	0.8894
	Other	0.0054	0.000000000000000000
	PhD	0.00545	0.9858
**Cadre**			
	Administrative staff	Ref	
	Caregivers	1.637643	**0.0128**
	Environmental health staff	1.911255	**0.0105**
**Sources of information**			
	**Government**	**1.6906**	**0.00364**
	News	0.9903	0.9429
	International	0.9805	0.8797
	**Social media**	**0.7543**	**0.0295**

	**Continuous medical fora**	**0.7090**	**0.0224**
	Journals	0.7869	0.1273

**Target variable**	**Predictor variable**	**COR (C.I.)**	**p-value**
**Practice**			
**Sex**			
	Female	Ref	
	Male	0.8609	0.4180
	Prefer not to say	2.7526	0.3143
**Age**			
	18-24	Ref	
	25-29	0.3574193	0.3113
	age30-34	0.5129216	0.5115
	age35-39	1.0127823	0.9901
	age40-44	0.8247799	0.8511
	age45-49	0.9600965	0.9692

	age50-54	0.5395713	0.5610
	age55-59	0.5129172	0.5246
**Work place**			
	Level 1	0.2568	0.0649
	2	2.2656	0.2365
	3	0.9365	0.9145
	4	0.5866	0.3702
	5	2.0200	0.2585
	Sub-county	0.6922	0.5818
	County official	Ref	
**Education**			
	Bachelors	Ref	
	Certificate	1.649	0.1373

	Diploma	1.9839	**0.0012**
	Masters	1.6459	0.1517
	Other	0.3482	0.0522
	PhD	3.2190	0.2493
**Cadre**			
	Administrative staff	Ref	
	Caregivers	2.2124	**0.0005**
	Environmental health staff	1.2154	0.5231
**Sources of information**			
	Government	1.7868	**0.0042**
	News	0.5858	**0.00519**
	International	0.7953	0.2019
	Social media	0.3832	**0.000000196**

	Continuous medical fora	0.7766	0.2221
	Journals	0.7135	0.1112
