# Supplementary material for: Knowledge, attitudes and practices towards COVID-19 among healthcare workers: A cross-sectional survey from Kiambu County, Kenya
Source: PLoS One. 2024 Mar 12;19(3):e0297335. doi: 10.1371/journal.pone.0297335 (PMC10931472; doi:10.1371/journal.pone.0297335)
Supplement: S2 Table — Additional information regarding the study participants. (PDF) [file pone.0297335.s003.pdf]

S3 Table. Table of breakdown of Socio-demographic characteristics of caregivers, administrative staff and environmental health staff. Additional information regarding the study participants.

Variable		All [Number (%)]	Caregivers	Administrative staff	Environmental health staff
Online/hard copy	Online	129 (29.0%)	90 (69.8%)	19 (14.7%)	20 (15.5%)
	Hard copy	315 (71.0%)	232 (75.1%)	47 (15.2%)	30 (9.7%)
Sex		N = 428	N = 309	N = 65	N = 50
	Female	276 (64.5%)	207 (67.0%)	37 (56.9%)	30 (60.0%)
	Male	144 (33.6%)	94 (30.4%)	28 (43.1%)	20 (40.0%)
	Prefer not to say	8 (1.9%)	8 (2.6%)	0 (0%)	0 (0%)
Age		N= 441	N = 321	N= 66	N=50
	20-24	5 (1.1%)	3 (0.9%)	0 (0%)	2 (4%)
	25-29	71 (16.1%)	44 (13.7%)	21 (31.8%)	6 (12%)
	30-34	89 (20.2%)	68 (21.2%)	13 (19.7%)	7 (14%)
	35-39	**91 (20.5%)**	**69 (21.5%)**	**11 (16.7%)**	**10 (20%)**
	40-44	79 (17.9%)	64	6	7

			(19.9%)	(9.1%)	(14%)
	45-49	47 (10.7%)	30<br>(9.3%)	7<br>(10.6%)	10<br>(20%)
	50-54	27 (6.1%)	18<br>(5.6%)	5<br>(7.6%)	4<br>(8%)
	55-59	32 (7.3%)	25<br>(7.8%)	3<br>(4.5%)	4<br>(8%)
Facility		N=424	N = 306	N = 64	N= 50
	Level 1	9 (2.1%)	0<br>(0%)	0<br>(0%)	9<br>(18.0%)
	Level 2	49 (11.6%)	47<br>(15.4%)	1<br>(1.6%)	1<br>(2.0%)
	Level 3	84 (19.8%)	57<br>(18.6%)	14<br>(21.9%)	13<br>(26.0%)
	Level 4	**119 (28.1%)**	**80<br>(26.1%)**	**26<br>(40.6%)**	**12<br>(24.0%)**
	Level 5	117 (27.6%)	100<br>(32.7%)	13<br>(20.3%)	3<br>(6.0%)
	Sub-county official	29 (6.8%)	15<br>(4.9%)	5<br>(7.8%)	8<br>(16.0%)
	County official	17 (4.0%)	7<br>(2.3%)	5<br>(7.8%)	4<br>(8.0%)
Education		N = 441	N = 321	N = 66	N = 49
	Certificate	43 (9.8%)	55<br>(17.1%)	12<br>(18.2%)	16<br>(32.0%)
	Diploma	**263 (59.6%)**	**15<br>(4.7%)**	**35<br>(53.0%)**	**14<br>(28.0%)**

	Bachelors	84 (19.0%)	212 (66.0%)	17 (25.8%)	11 (22.0%)
	Masters	40 (9.1%)	31 (9.7%)	2 (3.0%)	6 (12.0%)
	PhD	6 (1.4%)	2 (0.6%)	0 (0%)	0 (0.0%)
	Other	5 (1.1%)	6 1.9%	0 0%	3 6.0%
Knowledge sources					
	Official government sites	**349 (78.6%)**	**256 (79.5%)**	**48 (72.7%)**	**41 (82.0%)**
	News media	254 (57.2%)	184 (57.1%)	38 (57.5%)	30 (60.0%)
	International health sites	223 (50.2%)	171 (53.1%)	23 (34.8%)	27 (54.0%)
	Social media sites	192 (43.2%	137 (42.5%)	29 (43.9%)	24 (48.0%)
	Continuous medical fora	95 (21.3%)	66 (20.5%)	10 (15.1%)	19 (38.0%)
	Medical journals	80 (18.0%))	62 (19.2%)	8 (12.1%)	10 (20.0%)
Number of sources					
	None	18 (4.1%)	14 (4.3%)	1 (1.5%)	1 (2.0%)
	One	**153 (34.5%)**	**108 (33.5%)**	**29 (43.9%)**	**14**

					**(28.0%)**
	Two	41 (9.2%)	26<br><br>(8.07%)	8<br><br>(12.1%)	7<br><br>(14.0%)
	Three	81 (18.2%)	63<br><br>(19.6%)	12<br><br>(18.1%)	6<br><br>(12.0%)
	Four	78 (17.6%)	59<br><br>(18.3%)	9<br><br>(13.6%)	8<br><br>(16.0%)
	Five	35 (7.9%)	21 (6.5%)	3 (4.5%)	11 (22.0%)
	Six	38 (8.6%)	31 (9.6%)	4 (6.0%)	3 (6.0%)
