# Supplementary material for: Knowledge, attitudes and practices towards COVID-19 among healthcare workers: A cross-sectional survey from Kiambu County, Kenya
Source: PLoS One. 2024 Mar 12;19(3):e0297335. doi: 10.1371/journal.pone.0297335 (PMC10931472; doi:10.1371/journal.pone.0297335)
Supplement: Attachment — Submitted filename: Response to Reviewers.docx [file pone.0297335.s004.docx]

**RESPONSE TO REVIEWERS**

1. Is the manuscript technically sound, and do the data support the conclusions?

The manuscript must describe a technically sound piece of scientific research with data that supports the conclusions. Experiments must have been conducted rigorously, with appropriate controls, replication, and sample sizes. The conclusions must be drawn appropriately based on the data presented.

- Reviewer #1: Yes
- Reviewer #2: Partly

**Here, we have reviewed the manuscript for the soundness and ensured that the data supports our conclusions**

2. Has the statistical analysis been performed appropriately and rigorously?

- Reviewer #1: Yes
- Reviewer #2: No

**We have taken note of Reviewer #2’s comment and have revised our statistical analysis to add to the rigor of our methods and results.**

3. Have the authors made all data underlying the findings in their manuscript fully available?

Reviewer #1: Yes

Reviewer #2: No

**Reviewer #2, Thank you for your observation regarding the availability of the data underlying our manuscript's findings. Our data was included as Supplementary Data S1.**

4. Is the manuscript presented in an intelligible fashion and written in standard English?

PLOS ONE does not copyedit accepted manuscripts, so the language in submitted articles must be clear, correct, and unambiguous. Any typographical or grammatical errors should be corrected at revision, so please note any specific errors here.

Reviewer #1: Yes

Reviewer #2: No

**We took heed of Reviewer #2’s comment and have revised our manuscript for clarity and conciseness.**

5. Review Comments to the Author

Please use the space provided to explain your answers to the questions above. You may also include additional comments for the author, including concerns about dual publication, research ethics, or publication ethics. (Please upload your review as an attachment if it exceeds 20,000 characters)

***Dear Reviewer #1,***

***Thank you for your thoughtful and detailed feedback on our manuscript. We sincerely appreciate the time and effort you dedicated to reviewing our work. We have carefully considered each of your points, and your insights have been invaluable in enhancing the quality and clarity of our research.***

***Please find our responses below.***

**Reviewer #1:**

Abstract:
• Their objective does not center on exploring the KAP. The term "evaluating" suggests an assessment of interventions, treatments, or comparisons between pre and post studies.

**We acknowledge your concern about the term "evaluating" and have revised it to "assessing" to better align with our objective. Thank you for pointing this out.**

• They utilized multivariate logistic regression to adjust for other variables and estimate the adjusted odds ratio (AOR) associated with a 95% confidence interval. It might be better to incorporate these details in the methods section.

**We have since revised our data analysis plan and have included it in the abstract as “Ordinal logistic regression”.**

• Instead of presenting p-values as "p-value <0.001," it would be clearer to state them as "p=xxxe-10."

**Your suggestion regarding the presentation of p-values has been duly noted and implemented. We now express them fully for better clarity.**

• Could you provide information on the number of healthcare workers with good/bad practices?

**We appreciate your observation regarding the need for information on healthcare workers with good/bad practices. We have revised the abstract to include this important detail.**

• In conclusion, the authors did not mention the associated factors that need improvement for raising awareness.

**We apologize for the oversight regarding the associated factors for raising awareness. Your feedback has prompted us to revise our conclusion, incorporating recommendations in line with the study findings.**

Methods:
• The authors briefly mention the study design, but a more detailed explanation is necessary.

**Thank you for pointing out the need for a more detailed explanation of our study design. We have provided additional information to ensure a comprehensive understanding.**

• The sample size calculation provided is quite general and lacks important information, such as the proportion (p) of healthcare workers in the study. A 50% proportion is not appropriate for this type of study. Based on previous research, the authors should calculate the sample size specifically for healthcare workers (HCWs).

• It is stated that this is an online survey, and the authors mention that the survey was conducted until they reached a sample size of 385 HCWs. However, it appears that this survey did not take the sample proportionally, and further clarification is needed.

**Your concerns about the sample size calculation have been addressed. We have recalculated the sample size specifically for healthcare workers based on relevant literature and clarified our approach for achieving a representative sample, as below:**

**We revised the sample size calculation based on a Ugandan study by Olum *et al.* (from which the questionnaire was adopted), where the proportion of sufficient KAP was 69%, 21% and 74%, respectively. We used these KAP scores to calculate the minimum required sample sizes for each outcome variable. We assumed a 95% confidence level (corresponding to a Z-score of 1.96) and a margin of error E of 5% (expressed as 0.05).**

**1. Knowledge: n = 323 HCWs.**

**2. Attitudes: n = 257 HCWs.**

**3. Practices: n = 307 HCWs.**

**To further enhance the representativeness of the sample, the weights of the specific cadres were targeted to mirror the population, thus, caregivers were to constitute 78% of the sample, administrative staff approximately 12.1%, and environmental health health 9.7% of the sample size.**

**In our study, 438 HCWs responded.**

Results:
• In lines 146-147, it would be helpful to provide the number of indicators and the total score of knowledge for better clarification.

**We have revised the discussion to provide the number of indicators and the total score of knowledge for better clarification, as suggested.**

• In line 189, it is not possible for the 95% confidence interval of the odds ratio (OR) to be negative (-0.05 to 0.15).

**Your observation about the negative 95% confidence interval of the odds ratio has been rectified. We have since revised our data analysis to ensure accurate reporting of the results and ensure none of the reported ORs have a negative sign.**

• The study suggests an inverse association between practices and attitudes with knowledge. Is this interpretation accurate?

**Regarding the interpretation of the inverse association between practices and attitudes with knowledge, we have reviewed the findings and confirmed the accuracy of our interpretation. This has been elaborated upon in the discussion section.**

Discussion:
• In lines 241-244, the authors suggest that there were more gaps in knowledge and practices among HCWs. They found that HCWs did not exhibit appropriate practices, yet they had sufficient knowledge. However, their data analysis did not support this observation, as it showed that HCWs with insufficient knowledge had good practices. These results appear contradictory.

**We appreciate your feedback on the potential contradiction in our interpretation of knowledge and practices among HCWs. We have revisited our discussion and made necessary adjustments to align with the study's results more accurately.**

• In line 277, a major limitation of the study was its cross-sectional, which prevented the authors from conducting a more in-depth exploration of the KAP items.

**Your point about the limitation of the study being cross-sectional has been duly noted and included in our limitations section.**

• While the authors presented a well-written conclusion, it would be beneficial to provide further clarification on the factors that need improvement and how to address them.

**Thank you for highlighting the need for further clarification on the factors requiring improvement and how to address them. We have revisited our conclusions and provided additional details to address this concern.**

***Once again, we are grateful for your thorough review and constructive feedback. Your input has significantly strengthened the quality and cohesiveness of our manuscript.***

***Dear Reviewer #2,***

***We appreciate your thoughtful feedback and valuable suggestions regarding our manuscript. Your insights have been extremely instrumental in improving the clarity and quality of our work. We have carefully considered each of your points and made the necessary revisions to address your concerns. We sincerely thank you for your time in reviewing this paper as it is our belief that your comments have greatly elevated the quality of our work.***

***Please find our responses below.***

**Reviewer #2:**

Title
The title is not easy to understand for the readers

**We understand your concern about the readability of the title. Based on your feedback, we have revised the title to make it more straightforward and accessible to readers: "Knowledge, Attitudes, and Practices towards COVID-19 among Healthcare Workers: A Cross-Sectional Survey from Kiambu County, Kenya." We hope this new title is more comprehensible.**

Abstract
• Background is well written

**Thank you for this encouraging comment.**

• The study period (May to August) is not consistent with the mentioned period in the study design on page 4. The questionnaire was used to collect data not for determining associated factors of KAP. Simple and multiple logistic regression, not multivariate logistic regression. See also comments in the methods and materials section.

**We appreciate your attention to detail regarding the study period and the use of the questionnaire. We have made the necessary adjustments to accurately reflect the study period in all instances** **as 11^th^ March 2021 to 12^th^ August 2021. Thank you for the astute correction regarding the questionnaire and data analysis, we have revised the statement to read “A structured questionnaire was used to explore the factors associated with COVID-19-related KAP”. We have since revised our approach to data analysis as ordinal logistic regression and we have indicated the same in the abstract.**

• Results should be revised according to the comments raised in the methods and materials sections

**We have revised the results section in alignment with the updated data and methodology.**

• ‘Messenger’ word should be rephrased; authors used this term in the whole manuscript.

**Your feedback on the use of the term "messenger" has been duly noted, and we have rephrased it throughout the manuscript for consistency and clarity.**

Keyword
• Most of the keywords are overlapped with the title of this study

**We understand your concern about the overlap between keywords and the title. To address this, we have revised the keywords to ensure they are distinct from the title: Knowledge, attitudes, practices; Healthcare workers; pandemic; Health communication; efficacy beliefs.**

Introduction
• The introduction section is not shaped and written as required for scientific research

**We appreciate your feedback on the introduction section. We have restructured and rewritten the introduction to meet the scientific research standards.**

•The authors did not consider quality points to highlight why they conducted this study, and about research gaps

**The revised introduction now includes quality points highlighting the reasons for conducting the study.**

•Need a comprehensive review of relevant literature

**The revised introduction now includes a comprehensive review of relevant literature. Thank you.**

• The objective of this study is not clearly mentioned.

**We revised the objective of the study at the end of the Introduction section “This study sought to determine the KAP of HCW towards COVID-19 in Kiambu county, Kenya.”.**

•What are the implications of the findings of this study

**The revised introduction now includes a paragraph in the introduction section to cover this, as below:**

**“**However, given the paramount role of HCWs in pandemic response, what they know, feel and do becomes crucial in the face of a public health emergency. This situation is further complicated by the “infodemic” that paralleled the COVID-19 pandemic [15], and by a supply chain crisis that affected the availability of PPEs for adhering to proper protocols [16]. To avert the higher rate of being infected among HCWs, equipping them with good knowledge and practice is imperative, especially in countries with already low health worker-to-population ratio [17]. Thus, the KAP of HCW towards COVID-19 were critical to the success of the overall COVID-19 response.”

Methods and Materials
• The definition of the study population is not clear. It is also not clear, who are actually HCWs in this study or the way authors defined HCW, required quality modification.

**The above two observations have been noted, and thus the definitions have been revised according to the categories used in our study. In this study, a HCW included any person involved in the provision of health services to a user or those who are on facility grounds employed by the facility. At the time of the study, the county of Kiambu was served by a total of 3700 HCWs. These HCWs were divided into caregivers, administrative staff and environmental health workers, and details of each category can be found below.**

**1.	Caregivers included all HCWs who interact with patients directly. They included: medical officers, consultants, nurses, clinical officers, dental officers, dental technologists, pharmacists, pharmaceutical staff, laboratory staff, orthopedic technologist, nutritionists, radiographers, physiotherapists and mortuary attendants.**

**2.	Administrative staff included HCWs who do not interact with patients directly. They included: health administrative officers and staff, health-supportive staff, medical engineering technologists, health records & information officers, medical social workers, ambulance drivers, and HIV testing services staff.**

**3.	Environmental health workers consisted of public health staff, such as community health volunteers, health promotion officers, and public health officers/community health officers**

• According to the nature of the study setting and study population, the sample size determination and sampling technique are inappropriate. The sampling on a volunteer basis is not scientifically robust.

**In this study, we employed voluntary purposive sampling. While this approach would not be the best, we had to specifically target HCWs who then had to consent to participate in the study. This approach has been used previously in similar KAP studies, such as one by** Mark et al., 2022**, and was adopted here.**

• It is confusing, what type of questionnaire the authors used to collect the data: structured/open-ended/semi-structured? How could a questionnaire be structured and open-ended simultaneously? Probably, the authors considered a semi-structured research tool

**Thank you for this observation. It is correct that we employed a semi-structured questionnaire to collect data, but this manuscript only details the results of the structured questionnaire. We have corrected the manuscript accordingly.**

• The authors did not mention how they checked the reliability, validity, and consistency of their data and questionnaire

**This is a good observation and has been addressed in the manuscript in the section headed as “Questionnaire”. The main additions can be found below:**

**We employed a questionnaire from Olum et al.**(Olum et al., 2020) **for this study. This questionnaire was constructed as such:**

**1.	Knowledge was assessed using a 11-item questionnaire adapted from Zhong et al., and modified to suit HCWs, each correct answer weighing one point. According to Zhong et al., this was a reliable knowledge scale for adoption as it possessed a Cronbach’s alpha score of 0.71** (Zhong et al., 2020)**.**

**We adopted this Knowledge scale for our study, however we added three context-specific questions regarding the location of Kiambu county’s COVID-19 isolaton centre, handling a suspected COVID-19 case and data entry for suspected COVID-19 cases. These questions were selected and reviewed by public health experts.**

**2.	Attitudes were assessed using 5 Likert-item questions that have been adopted from Goni et al. and modified by Olum et al. Goni et al.’s Cronbach’s alpha scale measurement for this was 0.77** (Goni et al., 2020)**.**

**We adopted this Attitude scale for our study, however we added six context-specific questions regarding how the national and county government structures were handling the pandemic at various stages.**

**3.	Practices were assessed using five Likert-item questions that have been developed from the WHO recommended practices. This was adopted fully from the Olum et al.**

**The alpha measurements for the scale subsets as used in our study were as follows: αk = 0.69, αa = 0.81 and αp = 0.61. In this study, a Cronbach’s alpha score of above 0.6 was considered adequate, similar to other KAP studies** (Gopalakrishnan et al., 2021; Majmundar et al., 2018; Mark et al., 2022; Sayili et al., 2022)**.**

• The authors did not mention what was the basis of the classification of the target variables

**This is duly noted. We have revised our study to use Bloom’s cutoff points as the basis of classifying our target variables into Good (>80%), Medium (60-79%) and Poor (<60%).**

• The authors did not check the bivariate association of the target variables with the predictor variables, so, how did they select probable predictor variables for multiple regression modeling? In the modeling, some potential predictors may be insignificant due to arbitrary selection of the predictors.

**Given this comment, we had revisited the analysis and revised the data analysis plan to conduct ordinal logistic regression given the application of Bloom’s cutoff points that divided each KAP target variable into Good, Medium and Poor.**

• The authors actually performed binary and multiple logistic regression but they mentioned “multivariate logistic regression”

**This is duly noted and has been modified according to our new analysis. Thank you.**

• How the authors selected the link function for logistic regression as they did not check the nature of the target variable

**Thank you for this observation. We have revisited our analyses and reorganized our target variable according to the Bloom’s cutoff points for KAP studies and have organized our outcomes, such as type of knowledge into: Good, Medium and Poor. The link function was chosen based on the changes in the cumulative probabilities: the “probit” link function for gradual changes (as noted with the knowledge and attitude types) and the complementary log-log link function for practice, for which the cumulative probabilities increased from 0 slowly and then rapidly approached** (Chapter 12 Ordinal Logistic Regression | Companion to BER 642: Advanced Regression Methods, n.d.).

Results
• The results should be revised according to the comments in the methodology section

**This was duly noted and the entirety of the results section has been revised thoroughly.**

Authors should also consider the following specific observations:
• The results are poorly written and presented

**Thank you for this observation. We have rewritten the entirety of the results section again for enhanced clarity.**

**- We have thoroughly reviewed and refined the presentation of our results. The results of the ordinal logistic regression now include all the KAP outcomes and only the significant variables. This approach enhances the clarity of our findings and makes the presentation more precise and accessible to readers.**

**We hope these revisions address your concerns about the clarity and presentation of our results.**

• Tables are too long

**Thank you for your observations regarding the presentation of the results. We greatly value your feedback and have taken your comments into serious consideration. Based on your suggestions, we have made the following revisions to address your concerns:**

**1. Tables Length:**

**- We acknowledge (and share) your concern about the length of the tables. To address this, we have revised Table 1 to include only the socio-demographic characteristics of the sample. Cadre proportions have been mentioned in the narrative section of the results, ensuring a more concise presentation of information. We have also included the data pertaining to the specific cadres as Supplementary material S3.**

**- Additionally, we have truncated the other tables where possible, ensuring a more focused and streamlined presentation of the data.**

**- We have also included the crude odds ratio results and the p-values as Supplementary material S2.**

• Authors unnecessarily mentioned too long questions in the text

**We have revised the results section to eliminate references to long questions, ensuring a more concise and reader-friendly presentation of our findings.**

• Authors used ‘n’ for indicating sample size in the methodology section; in the result section it is confusing about the notation ‘n’ and ‘N’

**We have rectified the use of 'n' and 'N' to accurately represent whether it refers to the sample or population size, ensuring clarity in both the methodology and results sections.**

• In Tables 4, 6, and 8, the authors provided a column ‘p-value’. P-value of which estimates: crude odds ratio (COR) or adjusted odds ratio (AOR)? If it indicates AOR, then where is the significance value of the COR estimates? Without significance value, how these results could be interpretable?

**We thank you again, for this invaluable comment and apologize for its omission. In the revised manuscript, we have included the coefficient, AOR (with confidence intervals in brackets) and p-values only.**

• It is clear from Tables 4, 6, and 8, that the authors performed simple logistic regression for all predictor variables as well as performed multiple logistic regression including all the predictor variables in the model, Why? Why did the authors consider the predictors with insignificant COR in the multiple logistic regression modeling?

**We thank you again, for this invaluable comment. We have revised the manuscript according to the refined results of ordinal logistic regression. For this analysis, we only included predictors with COR with p-values less than < 0.05 in our initial and final fitted models.**

• Estimates and p-values are not presented in a unique and standard format.

**The estimates and p-values in our results section have been restructured to adhere to a standard format, enhancing the consistency and readability of the presentation.**

• The second column of Tables 4, 6, and 8 should be renamed as ‘Label’
**Due to the restructuring of our analysis, the specific tables you referred to no longer exist. Nevertheless, we appreciate your suggestion, which has guided our approach in creating clearer and more organized tables. We are grateful for the direction.**

• In Table 4, the authors provided COR for attitude and practice considering these as predictor variables of knowledge. In principle, how attitude and practice could be the predictors of knowledge? Is there any basis or reference against this issue? If yes, why there is no adjusted odds ratio for these factors? Probably, the authors considered it wrongly. Authors should rethink and redesign

**We have since rethought and redesigned our analysis of our data to ordinal logistic regression. We have only considered the independent variables as predictors for each KAP dependent variable. These variables were chosen only if they had a crude odds ratio COR of less than p < 0.05.**

• In Tables 6 and 8, the authors did not consider the knowledge level as a predictor variable for attitude and practice, why?

**We have since rethought and redesigned our analysis of our data to ordinal logistic regression. This approach ensures a more accurate representation of the relationship between predictor variables and outcomes, addressing the concerns you raised regarding the inclusion of attitude, practice, and knowledge levels as predictors.**

• Is the result of correlation measurement between knowledge and practice correct? If yes, does this type of relationship exist in reality? The authors did not discuss the reason for this result in the discussion section. What would be the implications of this finding?

**In response to your query, we did conduct Spearman’s rank correlation for the non-normally distributed knowledge scores against the practice scores. The analysis indeed revealed an inverse relationship between knowledge and practice, which was statistically significant. However, we acknowledge that our discussion of this relationship in the manuscript was not as clear and comprehensive as it should have been.**

**Your feedback has prompted us to reevaluate our approach, and we have decided to restructure the discussion section into distinct segments. Specifically, we will now have separate sections dedicated to discussing the knowledge, attitude, and practice findings individually. Additionally, we will include a dedicated section to explore the relationship between knowledge and practice in greater depth. This restructuring will enable us to thoroughly analyze the implications of this finding and provide a more detailed and nuanced discussion on this significant aspect of our research.**

**We are genuinely grateful for your astute observation, which has guided us to enhance the clarity and depth of our discussion.**

Discussion
• The discussion section is not written as expected for a scientific article

**In response to your comments, we have diligently revised the discussion section to ensure a proper and accurate reflection of our methods and results.**

• The authors are suggested to revise the methodology and results according to the comments raised in the respective sections

**We have taken time to review both the methods and results section for congruency with our discussion.**

• They should rewrite this section by explaining the potential findings with proper supporting citations

**We have taken care to address the concerns raised in the respective sections and have provided a comprehensive explanation of our study findings. Additionally, we have strengthened our discussion by incorporating relevant literature to support our conclusions.**

• Authors are also suggested to add implications of the findings and limitation of the study

**To further enhance the quality of our manuscript, we have included a dedicated section discussing the implications of our study findings. Furthermore, we have outlined the limitations of our study, acknowledging the constraints and challenges we encountered during our research.**

References quoted:

*Chapter 12 Ordinal Logistic Regression | Companion to BER 642: Advanced Regression Methods*. (n.d.). Retrieved November 4, 2023, from https://bookdown.org/chua/ber642_advanced_regression/ordinal-logistic-regression.html

Goni, M. D., Naing, N. N., Hasan, H., Wan-Arfah, N., Deris, Z. Z., Arifin, W. N., Hussin, T. M. A. R., Abdulrahman, A. S., Baaba, A. A., & Arshad, M. R. (2020). Development and validation of knowledge, attitude and practice questionnaire for prevention of respiratory tract infections among Malaysian Hajj pilgrims. *BMC Public Health*, *20*(1), 1–10. https://doi.org/10.1186/S12889-020-8269-9/TABLES/5

Gopalakrishnan, S., Kandasamy, S., Abraham, B., Senthilkumar, M., & Almohammed, O. A. (2021). Knowledge, Attitude, and Practices Associated With COVID-19 Among Healthcare Workers in Hospitals: A Cross-Sectional Study in India. *Frontiers in Public Health*, *9*, 787845. https://doi.org/10.3389/FPUBH.2021.787845

Majmundar, A., Allem, J. P., Cruz, T. B., & Unger, J. B. (2018). The Why We Retweet scale. *PLoS ONE*, *13*(10). https://doi.org/10.1371/journal.pone.0206076

Mark, E., Udod, G., Skinner, J., & Jones, M. (2022a). Knowledge, attitudes, and practices [KAP] toward COVID-19: A cross-sectional study in the New York Metropolitan Area and California Bay Area. *PLOS ONE*, *17*(8), e0271212. https://doi.org/10.1371/JOURNAL.PONE.0271212

Mark, E., Udod, G., Skinner, J., & Jones, M. (2022b). Knowledge, attitudes, and practices [KAP] toward COVID-19: A cross-sectional study in the New York Metropolitan Area and California Bay Area. *PLoS ONE*, *17*(8 August). https://doi.org/10.1371/journal.pone.0271212

Olum, R., Chekwech, G., Wekha, G., Nassozi, D. R., & Bongomin, F. (2020). Coronavirus Disease-2019: Knowledge, Attitude, and Practices of Health Care Workers at Makerere University Teaching Hospitals, Uganda. *Frontiers in Public Health*, *8*. https://doi.org/10.3389/fpubh.2020.00181

Sayili, U., Siddikoglu, E., Pirdal, B. Z., Uygur, A., Toplu, F. S., & Can, G. (2022). The heat wave knowledge, awareness, practice and behavior scale: Scale development, validation and reliability. *PLoS ONE*, *17*(12 December). https://doi.org/10.1371/journal.pone.0279259

Zhong, B. L., Luo, W., Li, H. M., Zhang, Q. Q., Liu, X. G., Li, W. T., & Li, Y. (2020). Knowledge, attitudes, and practices towards COVID-19 among Chinese residents during the rapid rise period of the COVID-19 outbreak: a quick online cross-sectional survey. *International Journal of Biological Sciences*, *16*(10), 1745. https://doi.org/10.7150/IJBS.45221
